# UFMylation of SHBs increases protein stability by antagonizing ubiquitination and promotes virion production

**DOI:** 10.1128/jvi.01034-26

**Published:** 2026-09-22

**Authors:** Shuxiang Wu, Yilin Huang, Jing Xu, Jie Li, Xiaohuang Lin, Xinjian Lin, Xu Lin

**Affiliations:** 1 Key Laboratory of Gastrointestinal Cancer (Fujian Medical University), Ministry of Education 74551, Fuzhou, China; 2 Fujian Key Laboratory of Tumor Microbiology, Department of Medical Microbiology, Fujian Medical University 74551, Fuzhou, China; Wake Forest University School of Medicine, Winston-Salem, North Carolina, USA

**Keywords:** small HBV surface antigen, UFM1, UFMylation, hepatitis B virus, UFL1

## Abstract

Post-translational modifications orchestrate virus–host interactions, yet their roles in the hepatitis B virus (HBV) life cycle remain incompletely defined. The small hepatitis B surface antigen (SHBs)—the most abundant HBV protein—drives immune evasion, pathogenesis, and virion/subviral particle (SVP) production. Here, we identify UFL1, the E3 ligase of UFMylation, as a host factor that modifies and stabilizes SHBs by antagonizing its ubiquitination to potentiate HBV output and SHBs-driven pro-oncogenic activities. Using affinity purification-mass spectrometry, we discovered UFL1 as a SHBs interactor and validated the interaction by co-immunoprecipitation, confocal co-localization, and GST pull-down assays. *In vivo* and *in vitro* UFMylation assays demonstrated that UFL1 catalyzes SHBs’ UFMylation. Site-directed mutagenesis mapped the modification to K122; the K122R mutant failed to undergo UFMylation. Cycloheximide chase and ubiquitination assays showed that UFMylation at K122 stabilizes SHBs by antagonizing TRIM21-mediated K48-linked polyubiquitination and proteasomal degradation. Functionally, UFL1 enhanced SHBs-driven hepatocellular carcinoma cell migration and endothelial tube formation, whereas UFL1 knockdown produced opposite effects. In HBV-producing cells, UFL1 increased intracellular and extracellular HBsAg and HBV virions, with no effects on HBV RNA, indicating post-translational control of HBsAg and virion/SVP production. These findings establish SHBs as, to our knowledge, the first HBV protein substrate of UFMylation and reveal a UFL1-SHBs axis that promotes SHBs stability, pathogenic functions, and HBV particle output. Targeting UFMylation or the UFL1-SHBs interface offers a host-directed strategy to reduce HBsAg and virion production and may inform therapeutic approaches for HBV infection and HBV-related liver disease.

**IMPORTANCE:**

Chronic infection with hepatitis B virus (HBV) remains a major global health problem, in part because current treatments rarely reduce the viral surface protein that floods the blood and helps the virus persist. In this study, we asked how the most abundant viral surface protein—SHBs—is controlled inside cells. We discovered that UFL1 attaches a small “molecular tag” as UFM1 to this viral protein, and this tag protects the SHBs from the cell’s disposal system. As a result, the increased SHBs promotes behaviors linked to liver disease progression, and cells release more virion/SVPs. When we prevented this tagging event or changed a single amino acid on the SHBs so it could not be tagged, the protein became unstable and was degraded, and virus output dropped. Thus, to our knowledge, SHBs is the first identified HBV protein substrate of UFMylation, suggesting targeting the UFL1-SHBs interface may reduce HBsAg and virion production and inform HBV therapy.

## INTRODUCTION

Hepatitis B virus (HBV) remains a major global health challenge, with over 296 million people chronically infected and approximately 820,000 deaths each year (1). HBV is a hepatotropic virus with a partially double-stranded DNA genome organized into four overlapping open reading frames that give rise to pregenomic RNA (pgRNA), subgenomic RNAs, and seven viral proteins, including polymerase (Pol), e antigen (HBeAg), core antigen (HBc), X protein (HBx), and the three forms of surface antigens (HBsAg)—LHBs (large), MHBs (middle), and SHBs (small) with the molecular ratio of 1:1:4 (2). During HBV assembly, pgRNA and polymerase are packaged into nucleocapsids, where pgRNA is reverse-transcribed and subsequently generates relaxed circular DNA. Mature DNA-containing nucleocapsids are then enveloped by surface proteins to form infectious virions, which are secreted through the multivesicular body pathway (3). Although HBV is not directly cytolytic, its proteins contribute to hepatocarcinogenesis by promoting cell growth, evading apoptosis, modulating autophagy, inducing angiogenesis, facilitating metastasis, and shaping immune suppression (4). At each step, host factors critically influence HBV replication and pathogenesis, underscoring the importance of understanding host–virus regulatory interfaces (5). Thus, elucidating host-mediated regulation of viral protein stability is crucial for eradicating HBV and developing effective treatments for HBV-related liver diseases.

Among viral proteins, SHBs is the most abundant and plays central roles in immune evasion, particle secretion, and disease progression (6–8). Clinically, durable loss of HBsAg, especially SHBs, is considered a “functional cure,” highlighting the value of strategies that lower SHBs levels and output (9). The levels and functions of SHBs are modulated by host proteins via post-translational modifications, including glycosylation and ubiquitination (10, 11). N-linked glycosylation of SHBs is required for virion secretion (but not necessarily for subviral particles [SVPs]), and mutations within the major hydrophilic region can facilitate immune escape (12–14). In contrast to glycosylation, ubiquitin-mediated turnover pathways shape SHBs abundance: TRIM21 and OTUD4 regulate SHBs stability through K48-linked polyubiquitination and proteasomal degradation, thereby impacting virion/SVP production and associated biological activities (11). In addition to these modifications, whether there are other post-translational modifications in SHBs remains unclear and requires further clarification.

UFMylation is an emerging ubiquitin-like modification in which the ubiquitin-fold modifier 1 (UFM1) is conjugated to substrate proteins via its C-terminal Gly^83^ through an E1 (UBA5)–E2 (UFC1)–E3 (UFL1) enzymatic cascade;, DDRGK1 helps localize UFL1 to the endoplasmic reticulum (ER), facilitating its activity, while deconjugation is mediated by UFM1-specific proteases (UFSPs) (15). By altering protein stability, interactions, and localization, UFMylation regulates cell death, differentiation, and innate immunity, with essential roles in development and implications for cancer and viral infection (16). Across viruses, UFMylation acts bidirectionally, enhancing or restraining infection in a context-dependent way. For example, UFL1-dependent UFMylation of Dengue virus (DENV) proteins (NS2A, NS2B-NS3, and capsid) and Zika virus (ZIKV) proteins (NS2A and NS3) supports the assembly of infectious particles without altering translation or RNA replication, thereby increasing infectious yield (17). Epstein–Barr virus (EBV) co-opts UFL1 to UFMylate MAVS via the viral receptor BILF1, shunting MAVS into mitochondrial-derived vesicles for lysosomal degradation to dampen inflammasome activity and favor replication (18). Hepatitis A virus (HAV) leverages UFL1-mediated UFMylation of the ribosomal protein RPL26 to enhance viral RNA translation (19). Conversely, UFL1 can augment host antiviral defenses by stabilizing STING through UFMylation, thereby sustaining innate immune signaling (20), and by facilitating 14-3-3ε-dependent recruitment of RIG-I to MAVS to induce interferons during RNA virus infection (21). Thus, UFMylation is a bidirectional regulatory axis in host–virus interactions, with documented roles in flaviviruses, EBV, and HAV—but whether it influences HBV protein homeostasis, particle production, or pathogenesis has remained unclear.

Here, we set out to determine whether the UFMylation E3 ligase UFL1 directly modifies SHBs, to map the site of modification, and to define how UFMylation influences SHBs stability, HBV particle production, and SHBs-mediated disease-relevant cellular behavior. We find that UFL1 interacts with SHBs and supports UFMylation at lysine 122, which counteracts TRIM21-mediated K48-linked polyubiquitination and proteasomal degradation, thereby stabilizing SHBs. Functionally, UFMylation-driven stabilization of SHBs increases virion/SVP output and enhances SHBs-associated hepatoma cells’ migratory and angiogenic activities. Collectively, these results identify SHBs as, to our knowledge, the first HBV protein substrate of UFMylation and reveal a host-directed regulatory mechanism that may be exploited therapeutically to reduce HBsAg and virion production in HBV infection.

## RESULTS

### SHBs interacted with UFL1

**Fig 1:**
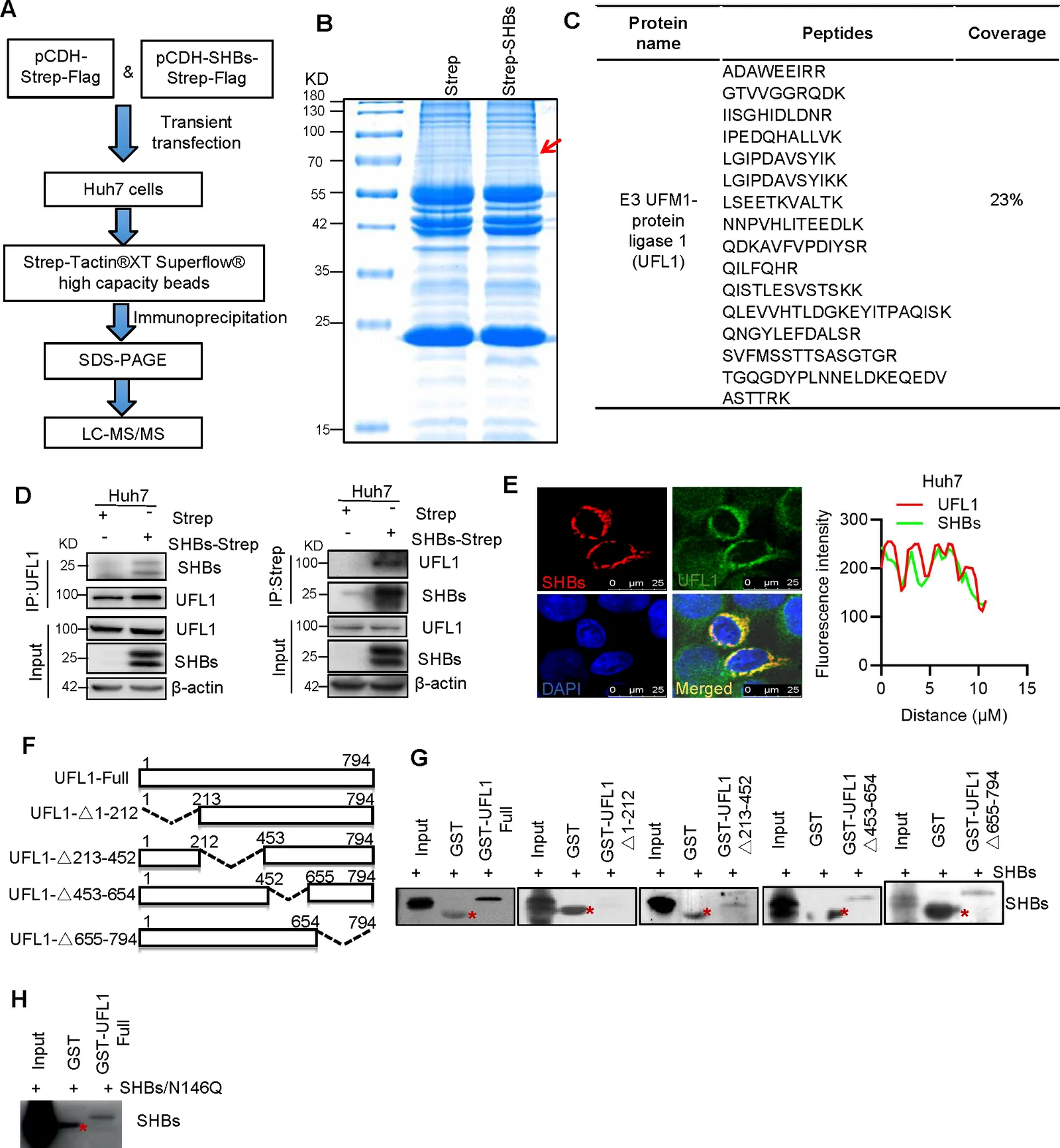
UFL1 interacted with SHBs. (**A**) Workflow for identifying SHBs-binding proteins by affinity purification-mass spectrometry. (**B**) SHBs pulldown products were resolved by 12% SDS-PAGE; Coomassie-stained bands near ~90 kDa were excised and subjected to LC-MS/MS. (**C**) UFL1 peptide coverage map detected by LC-MS/MS, with identified tryptic peptides mapped onto the UFL1 primary sequence. (**D**) Huh7 lysates from cells expressing SHBs-Strep or Strep were immunoprecipitated with anti-UFL1 (left) or Strep-Tactin (right) and immunoblotted for SHBs or UFL1, respectively. (**E**) Huh7 cells were transfected with a plasmid encoding SHBs-Strep, and then probed with anti-SHBs rabbit antibody and anti-UFL1 rat antibody, followed by incubation with secondary antibodies and DAPI. Colocalization was viewed by Leica SP8 laser confocal microscopy. Scale bars represent 25 μm. (**F**) A schematic representation of UFL1 and its various deletion mutants. (**G**) Purified GST and GST-fused UFL1 wild-type or deletion fragments (Δ1–212, Δ213–452, Δ453–654, and Δ655–794) were incubated with glutathione-Sepharose agarose beads overnight. Then, purified beads were incubated with equivalent *in vitro*-translated SHBs, and pulled-down protein complexes were subjected to western blotting detection using anti-SHBs antibodies; the asterisk indicates non-specific binding of the GST tag. (**H**) GST-fused full-length UFL1 was incubated with equivalent amounts of *in vitro*-translated SHBs/N146Q, and the bound proteins were analyzed by western blotting.

SHBs, a key structural component of virions and subviral particles (SVPs), has been linked to increased hepatocellular carcinoma risk (22). To identify host factors that regulate SHBs, we performed affinity purification coupled with liquid chromatography-tandem mass spectrometry (LC-MS/MS) in Huh7 cells expressing SHBs-Strep-Flag (Fig. 1A). Coomassie-stained SDS-PAGE bands with the size around 90 kDa were excised and analyzed (Fig. 1B), revealing several candidate interactors, including UFL1, in which 16 unique UFL1 peptides (~23% coverage) were identified by LC-MS/MS (Fig. 1C; Fig. S1A). Co-immunoprecipitation (Co-IP) verified interaction between UFL1 and overexpressed SHBs (Fig. 1D; Fig. S1B). Confocal microscopy showed cytoplasmic co-localization (Fig. 1E) of UFL1 and SHBs. GST pull-down demonstrated a direct interaction between GST-UFL1 and *in vitro*-translated SHBs, and amino acids 1–212 of UFL1 are crucial for its binding to SHBs (Fig. 1F through G; Fig. S1C). SHBs is a glycoprotein containing a single N-linked glycosylation site at N146, which is important for HBV virion secretion (23). To determine whether p24, gp27, or both forms efficiently bind UFL1, we generated a plasmid encoding the nonglycosylated SHBs/N146Q mutant. GST pull-down assays showed that UFL1 binds to nonglycosylated SHBs (Fig. 1H). These data established UFL1 as a bona fide SHBs-binding protein.

### SHBs was UFMylated by UFL1

**Fig 2:**
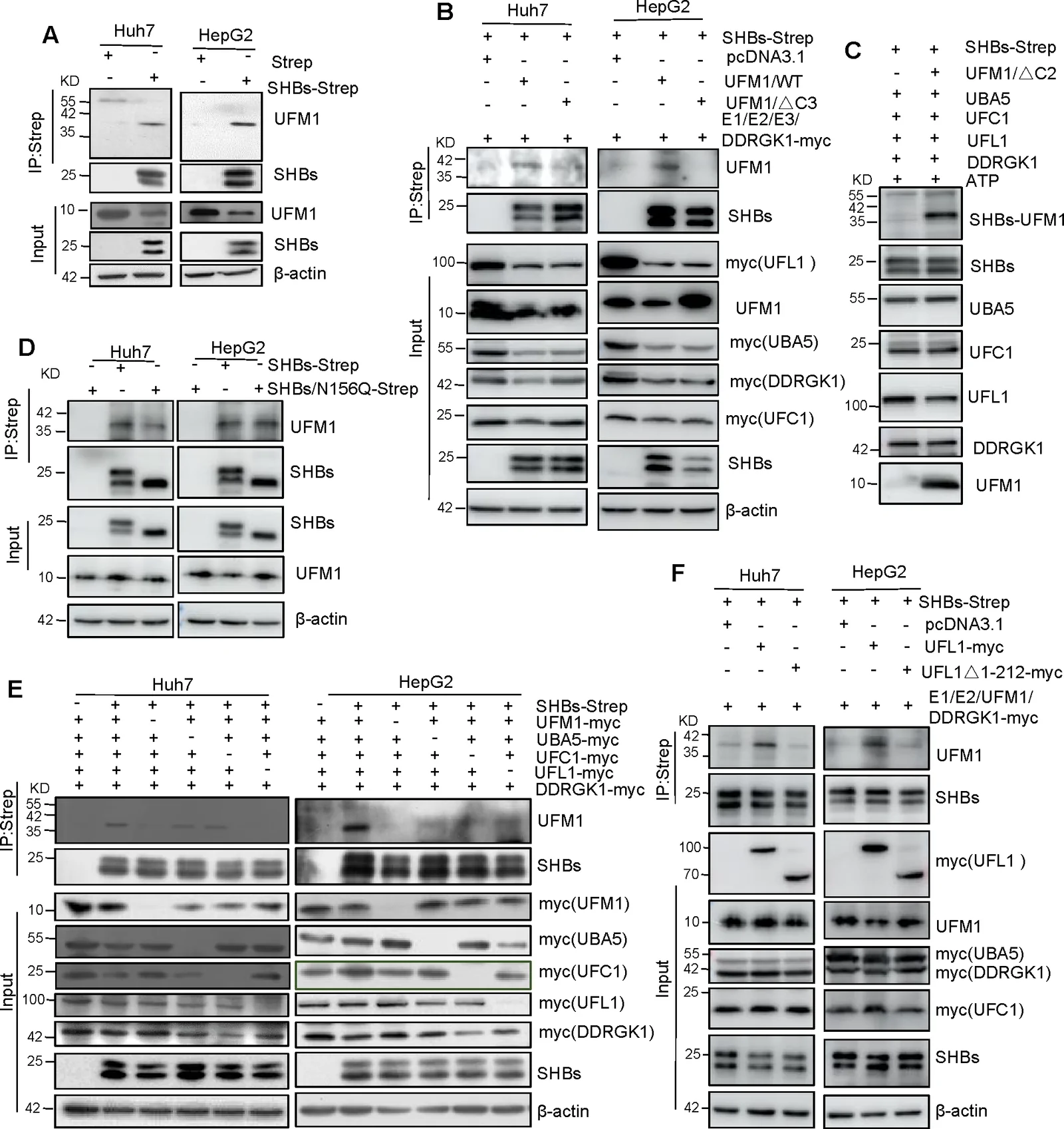
SHBs was UFMylated by UFL1. (**A**) SHBs UFMylation assessment. Huh7 or HepG2 cells co-transfected with plasmids encoding SHBs-Strep or Strep were treated with MG132 (20 µM) for 8 h, followed by *in vivo* UFMylation analysis. (**B**) *In vivo* UFMylation assay. Huh7 or HepG2 cells were co-transfected with plasmids encoding SHBs-Strep, myc-tagged UFMylation components, and myc-UFM1 or inactive form of myc-UFM1/ΔC3; 48 h later, UFMylation levels of SHBs was evaluated. (**C**) *In vitro* UFMylation assay. SHBs proteins, components, and the active form of myc-UFM1/ΔC2 were mixed and incubated in the presence of ATP and MgCl_2_ at 30°C for 90 min. UFMylation levels of SHBs in the mixture was evaluated. (**D**) Huh7 or HepG2 cells were transfected with plasmids encoding SHBs-Strep or SHBs/N146Q-Strep and treated with MG132 (20 µM) for 8 h, followed by *in vivo* UFMylation assays. (**E**) Effects of individual UFMylation component on SHBs UFMylation. SHBs UFMylation in Huh7 or HepG2 cells was assessed lacking each of the UFMylation components UFM1, UBA5, UFC1, or UFL1. (**F**) Huh7 or HepG2 cells were co-transfected with plasmids encoding SHBs-Strep, Myc-tagged UFMylation components, and either Myc-UFL1 or Myc-UFL1Δ1–212. SHBs UFMylation was analyzed 48 h after transfection.

Because UFL1 is the only known UFMylation E3 ligase (24), we tested whether SHBs is UFMylated by UFL1. In Huh7 or HepG2 cells expressing SHBs-Strep and treated with MG132, *in vivo* UFMylation assays detected immunoprecipitated UFMylated SHBs species (Fig. 2A). Co-expression of SHBs with UFMylation components, including E1 (UBA5)/E2 (UFC1)/E3 (UFL1)/DDRGK1, yielded robust SHBs UFMylation with wild-type UFM1 (UFM1/WT), but not with the unconjugated form of UFM1/ΔC3 lacking the C-terminal Gly^83^ residue (Fig. 2B). In a reconstituted *in vitro* system using SHBs-Strep purified from HEK293T cells, recombinant UFMylation components, and UFM1/ΔC2, an active form of UFM1 containing the C-terminal Gly^83^ residue, all purified from *Escherichia coli* Rosetta (DE3), we observed direct SHBs UFMylation (Fig. 2C). These results indicate that SHBs undergo UFMylation both in cells and *in vitro*. To determine whether glycosylated or nonglycosylated SHBs can be preferentially UFMylated, we transfected Huh7 and HepG2 cells with plasmids encoding SHBs-Strep or SHBs/N146Q-Strep and treated the cells with MG132. *In vivo* UFMylation assays showed that nonglycosylated SHBs can be UFMylated as well (Fig. 2D). Systematic omission of individual UFMylation components revealed that omission of UFM1, UBA5, UFC1, or UFL1 markedly impaired SHBs UFMylation (Fig. 2E). In contrast, neither overexpression of UFM1 nor UFL1 alone nor co-expression of UBA5, UFC1, UFL1, and DDRGK1 induced detectable SHBs UFMylation (Fig. S2A). To further investigate the role of DDRGK1 in SHBs UFMylation, we depleted DDRGK1 using specific siRNAs and found that DDRGK1 knockdown had no effect on SHBs UFMylation (Fig. S2B). To further define the role of UFL1, we transfected cells with plasmids encoding either wild-type UFL1 or a UFL1 mutant lacking amino acids 1–212. Cellular UFMylation assays showed that wild-type UFL1 promoted SHBs UFMylation, whereas the UFL1 Δ1–212 mutant failed to do so (Fig. 2F). These results indicate that UFM1, UBA5, UFC1, and UFL1 are required for SHBs UFMylation, whereas DDRGK1 is dispensable.

### UFL1 stabilized SHBs by antagonizing ubiquitination-dependent proteasomal degradation

**Fig 3:**
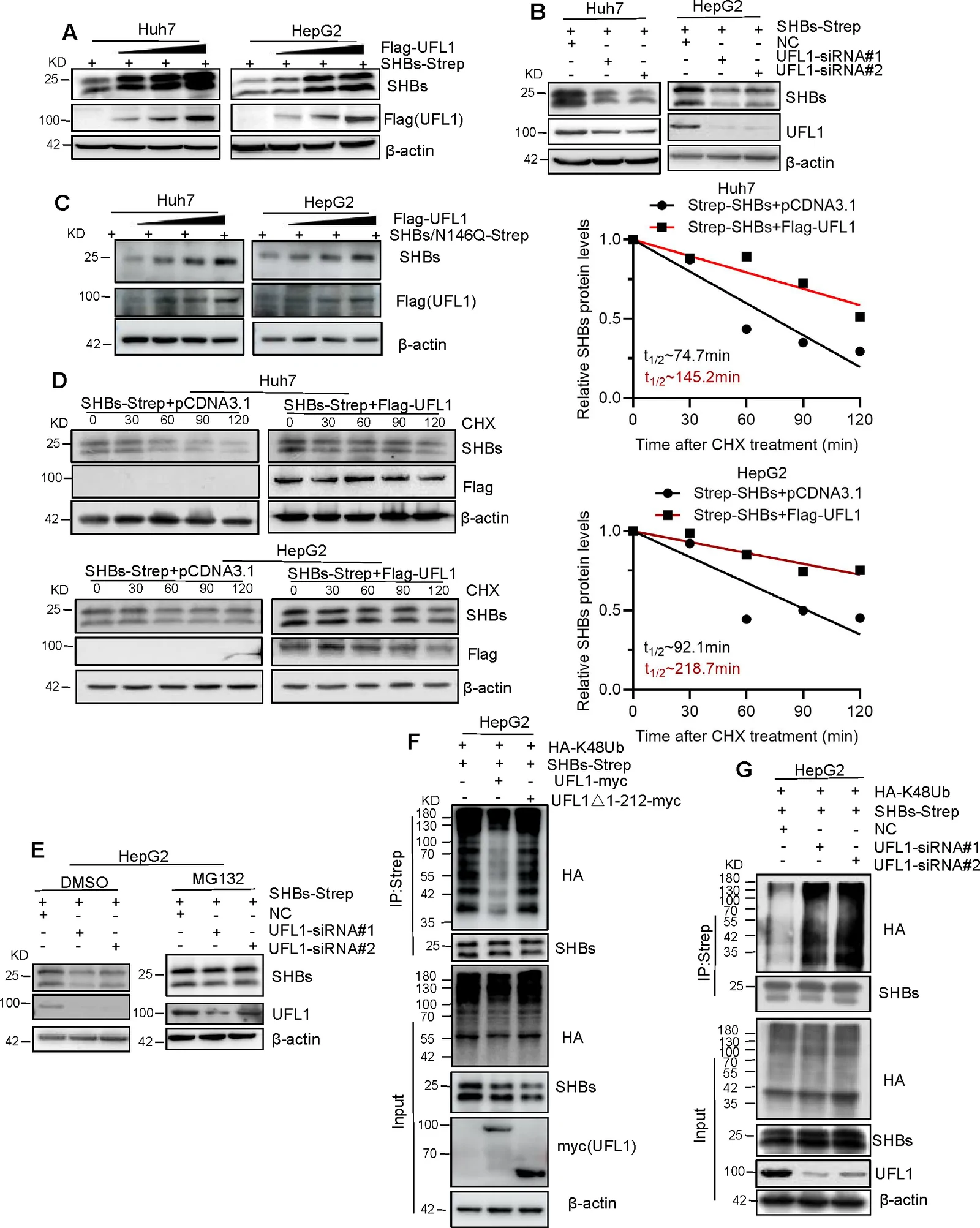
UFL1 stabilized SHBs by antagonizing ubiquitination-dependent proteasomal degradation. (**A**) Huh7 or HepG2 cells were co-transfected with plasmids encoding SHBs-Strep and increasing amounts of Flag-UFL1; the cell lysates were harvested and immunoblotted with specific antibodies against Flag (UFL1), β-actin, and SHBs. (**B**) Huh7 or HepG2 cells were transfected with plasmids encoding SHBs-Strep and siRNAs targeting UFL1 or NC; the cell lysates were assessed via western blot analysis. (**C**) Huh7 or HepG2 cells were co-transfected with plasmids encoding SHBs/N146Q-Strep and increasing amounts of Flag-UFL1, followed by western blot analysis. (**D**) Huh7 or HepG2 cells were co-transfected with plasmids encoding SHBs-Strep and Flag-UFL1 or the control vector for 40 h and treated with CHX (200 µg/mL) at 0, 30, 60, 90, and 120 min; the cell lysates were assessed via western blot analysis, with a graph depicting SHBs protein level. (**E**) HepG2 cells were co-transfected with plasmids encoding SHBs-Strep and UFL1-specific siRNAs and were treated with MG132 (20 µM) for 8 h; the cell lysates were analyzed by western blot analysis. (**F, G**) Effects of UFL1 or UFL1 Δ1–212 on the ubiquitination of SHBs. HepG2 cells were co-transfected with plasmids encoding SHBs-Strep, HA-K48UB, and Myc-UFL1 or Myc-UFL1 Δ1–212 (**F**), or UFL1-specific siRNAs (**G**), and treated with MG132 (20 µM) for 8 h; ubiquitination levels of SHBs were evaluated.

UFL1 mediates protein UFMylation to antagonize ubiquitination, thereby subsequently reducing degradation and enhancing stability (25). We next examined whether UFL1 modulates SHBs abundance. Co-expression of UFL1 increased SHBs in a dose-dependent manner in Huh7 and HepG2 cells (Fig. 3A), whereas siRNA-mediated UFL1 knockdown reduced SHBs (Fig. 3B). To determine whether UFL1 also regulates nonglycosylated SHBs, we co-expressed increasing amounts of UFL1 with the nonglycosylated SHBs/N146Q mutant in Huh7 and HepG2 cells. UFL1 similarly increased SHBs/N146Q protein levels in a dose-dependent manner (Fig. 3C), indicating that UFL1 enhances SHBs accumulation independently of N-linked glycosylation. We next investigated whether other components of the UFMylation machinery also regulate SHBs levels. Co-expression of UFM1, the E1/E2/E3 enzymes together with DDRGK1, or the complete UFMylation machinery (UFM1, UBA5, UFC1, UFL1, and DDRGK1) increased both intracellular and secreted SHBs levels in a dose-dependent manner (Fig. S3A through C), indicating that UFMylation promotes SHBs accumulation. To further investigate the role of UFL1 in the stability of SHBs, cycloheximide (CHX) chase assay was used, and the results demonstrated that UFL1 prolonged SHBs half-life (Fig. 3D). Building on our prior finding that SHBs is primarily turned over via a K48-linked polyubiquitination-dependent proteasome degradation pathway (11), we further explored the pathway through which UFL1 stabilizes SHBs. The results demonstrated that knockdown of UFL1 significantly decreased SHBs protein levels; this reduction was attenuated in the presence of MG132 (Fig. 3E), indicating that UFL1 stabilizes SHBs by reducing the proteasomal degradation. In addition, ubiquitination assays showed that overexpression of UFL1 reduced the polyubiquitination of SHBs, whereas the UFL1 Δ1–212 mutant failed to do so (Fig. 3F), whereas silencing UFL1 led to a significant increase in SHBs polyubiquitination (Fig. 3G). Together, these results indicated that UFL1 stabilized SHBs by suppressing its ubiquitination-dependent proteasomal degradation.

### UFMylation of SHBs occurred at lysine 122

**Fig 4:**
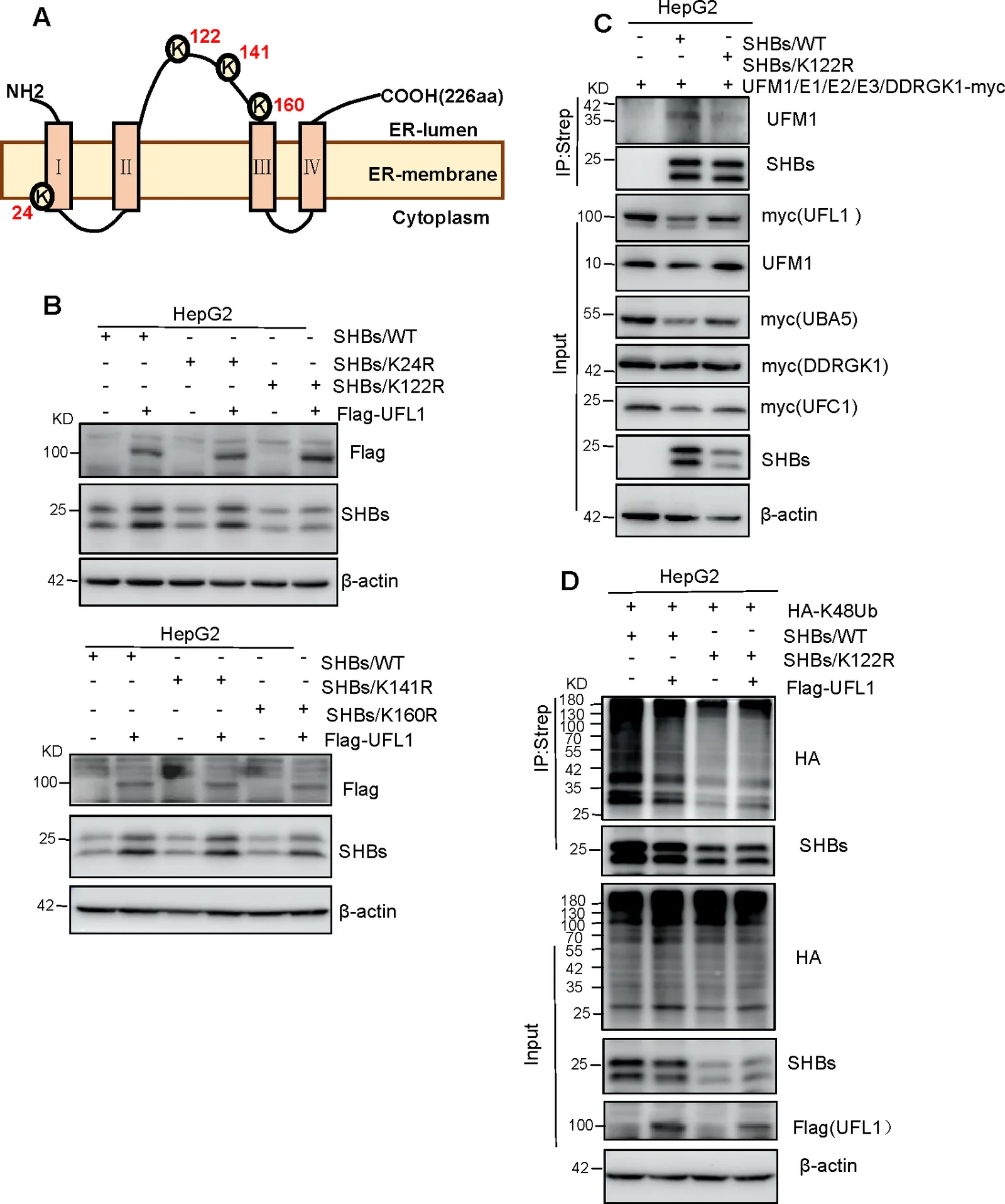
SHBs was UFMylated by UFL1 at Lys122. (**A**) Schematic representation of lysine residues in SHBs (protein ID: AAD16254.1, from Genotype B HBV, GenBank accession number: AF100309). (**B**) HepG2 cells were co-transfected with plasmids encoding Flag-UFL1 (or control vector) and wild-type SHBs or its various mutants (K residues at positions 24, 122, 141, and 160 were substituted with R); the cell lysates were assessed via western blot analysis. (**C**) HepG2 cells were co-transfected with plasmids encoding UFMylation components and SHBs or K122R mutant; UFMylation levels of SHBs were evaluated via UFMylation assay analysis. (**D**) HepG2 cells were co-transfected with plasmids encoding Flag-UFL1, HA-K48UB, and either wild-type SHBs or its K122R mutant, followed by an 8 h MG132 (20 µM) treatment, ubiquitination levels of SHBs were evaluated via ubiquitination assay analysis.

The genotype B clone used in this study contains four lysine residues in SHBs (Fig. 4A). We therefore generated lysine-to-arginine mutants at each site: K24R, K122R, K141R, and K160R, and co-transfected HepG2 cells with plasmids encoding Flag-UFL1 plus SHBs/WT-Strep or each mutant. The results demonstrated that UFL1 overexpression increased the abundance of SHBs/WT, K24R, K141R, and K160R, but had no effect on K122R, indicating that Lys122 was required for UFL1-dependent stabilization (Fig. 4B). Consistently, *in vivo* UFMylation assays showed that UFL1 enhanced UFMylated SHBs species for SHBs/WT but not for K122R, identifying Lys122 as the UFMylation acceptor site (Fig. 4C). Finally, ubiquitination assays demonstrated that UFL1 reduced SHBs polyubiquitination for SHBs/WT, whereas no reduction was observed for K122R mutant (Fig. 4D). Together, these data establish Lys122 as the UFL1-mediated UFMylation site that is necessary to antagonize SHBs polyubiquitination and thereby stabilize SHBs.

### UFL1 competed with TRIM21 to limit SHBs ubiquitination

**Fig 5:**
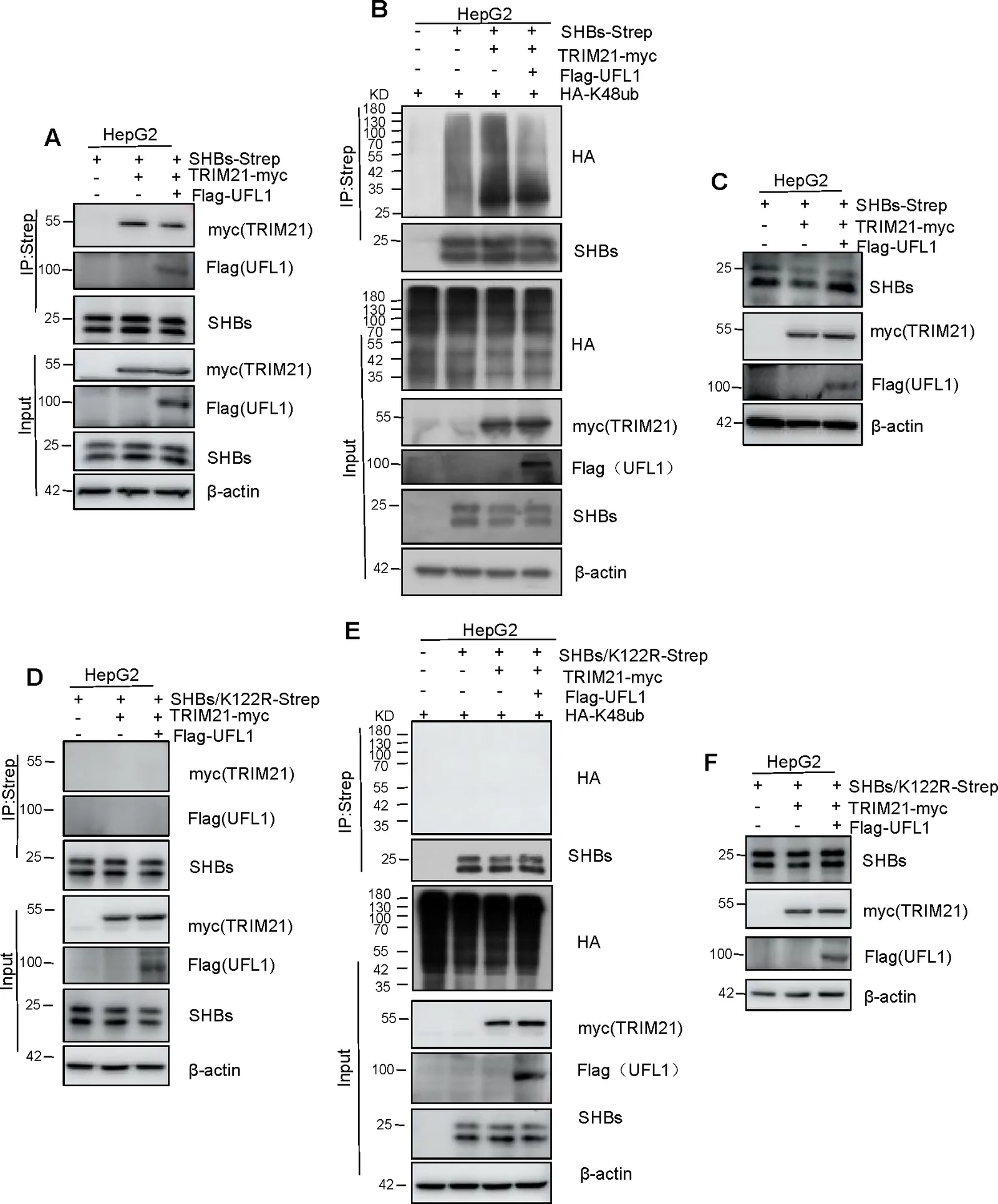
UFL1 decreased SHBs ubiquitination by competing with TRIM21. (**A**) HepG2 cells were co-transfected with plasmids encoding SHBs-Strep and TRIM21-myc (or control vector) and Flag-UFL1 (or control vector), and treated with MG132 (20 µM) for 8 h; the cell lysates were subjected to immunoprecipitation using Strep-Tactin and analyzed by immunoblotting. (**B**) HepG2 cells were co-transfected with plasmids encoding SHBs-Strep, HA-K48UB, TRIM21-myc (or control vector), together with or without Flag-UFL1, and treated with MG132 (20 µM) for 8 h; the ubiquitination levels of SHBs were evaluated via ubiquitination assay analysis. (**C**) HepG2 cells were co-transfected with plasmids encoding SHBs-Strep, TRIM21-myc (or control vector), together with or without Flag-UFL1; SHBs levels were evaluated via western blot analysis. (**D**) TRIM21 interaction with SHBs/K122R and its regulation by UFL1 were analyzed by Co-IP. (**E**) SHBs/K122R ubiquitination was analyzed in HepG2 cells co-transfected with plasmids encoding TRIM21-myc and HA-K48-UB, with or without Flag-UFL1, followed by MG132 treatment. (**F**) SHBs/K122R protein levels were analyzed by western blotting.

TRIM21 promotes K48-linked polyubiquitination and degradation of SHBs (11). We therefore tested whether UFL1 functionally counteracted TRIM21. Co-IP revealed that increasing UFL1 expression reduced the association between SHBs and TRIM21 (Fig. 5A). Functionally, TRIM21 overexpression increased SHBs polyubiquitination and lowered SHBs protein levels; both effects were mitigated by UFL1 (Fig. 5B and C). By contrast, the SHBs K122R mutant failed to interact with TRIM21, and UFL1 no longer influenced this interaction (Fig. 5D). Consistently, the K122R mutation abolished SHBs polyubiquitination and rendered SHBs resistant to TRIM21-mediated degradation, thereby eliminating the regulatory effect of UFL1 (Fig. 5E and F). These findings supported a competitive model in which UFL1 engagement with SHBs limited TRIM21 access, thereby decreasing polyubiquitination and stabilizing SHBs.

### UFL1 modulated SHBs-driven cell migration and angiogenic activity

**Fig 6:**
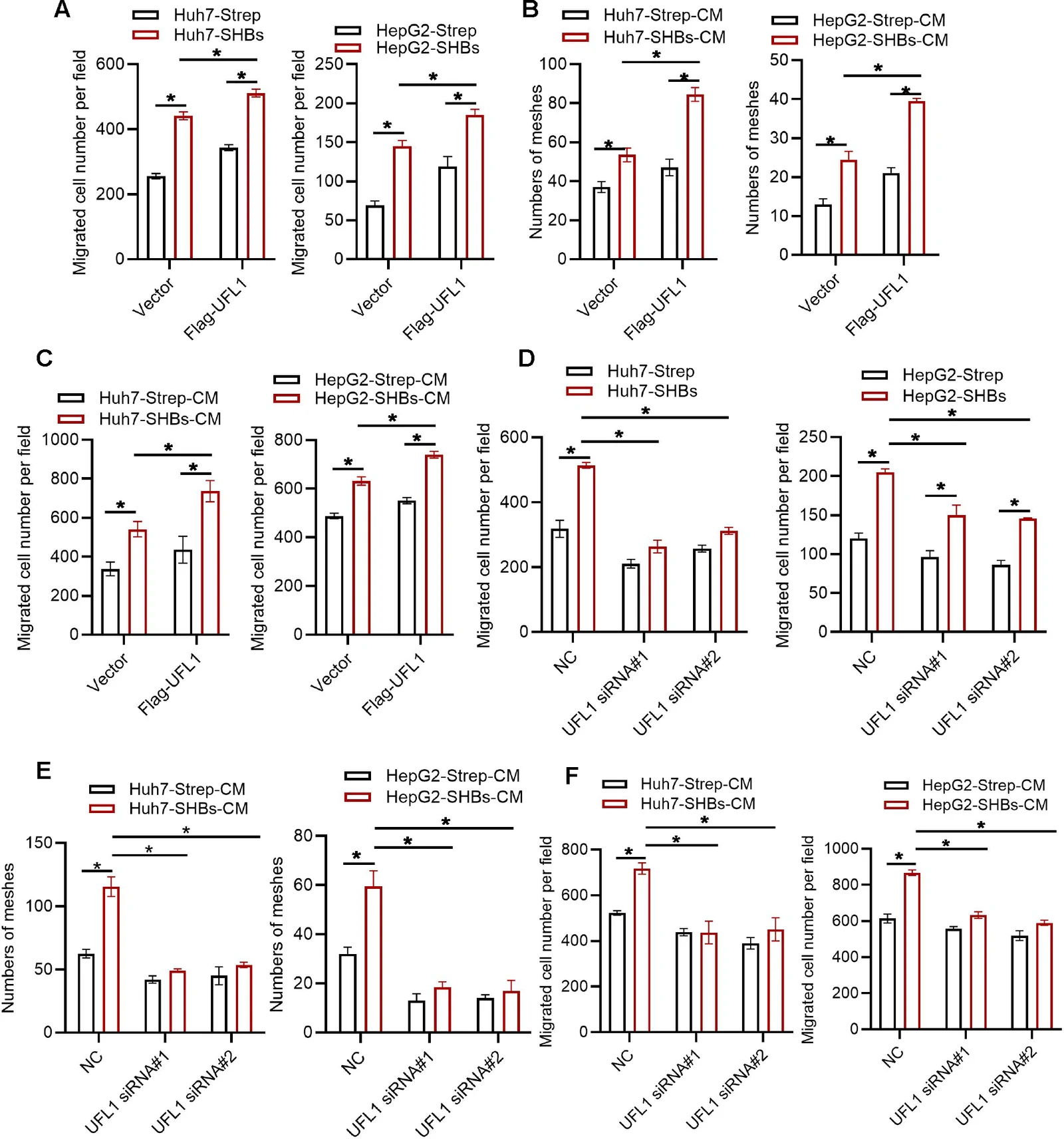
UFL1 modulated SHBs-driven migration and angiogenesis. (**A**) Huh7 and HepG2 cells were co-transfected with plasmids encoding SHBs-Strep (or Strep) and either Flag-UFL1 (or control vector). Transwell migration assays were performed to analyze the migration of cells. (**B, C**) Tube formation assays (**B**) and human vascular endothelial cell transwell migration assays (**C**) were carried out using EA.hy926 cells cultured in conditioned media (CM) derived from Huh7 or HepG2 cells that were co-transfected with plasmids encoding SHBs-Strep (or Strep) and Flag-UFL1 (or control vector). (**D–F**) Huh7 or HepG2 cells were co-transfected with plasmids encoding SHBs-Strep (or Strep) and UFL1-specific siRNAs (or negative control siRNA). The hepatoma cell transwell migration assays (**D**), tube formation assays (**E**), and EA.hy926 cell transwell migration assays (**F**) were carried out. Error bars represent SD; * indicates *P* < 0.05; quantification from ≥5 random fields is shown.

It has been demonstrated that SHBs not only enhances hepatoma cell metastasis but also induces angiogenesis triggered by VEGFA (6, 7). We next assessed whether UFL1 modulates SHBs-associated cellular behaviors. In SHBs-expressing Huh7 and HepG2 cells, UFL1 overexpression augmented SHBs-induced tumor cell migration in transwell assays (Fig. 6A). Conditioned media from cells co-expressing SHBs and UFL1 induced a more pronounced increase in both endothelial tube formation and migration of EA.hy926 cells than media from cells expressing SHBs alone (Fig. 6B and C). Conversely, siRNA-mediated UFL1 knockdown diminished SHBs-enhanced HCC cell migration and reduced endothelial tube formation and migration (Fig. 6D through F). Thus, UFL1 strengthens SHBs-driven promigratory and proangiogenic activities by stabilizing the SHBs protein.

### UFL1 modulated HBV virion output without altering HBV RNA abundance

**Fig 7:**
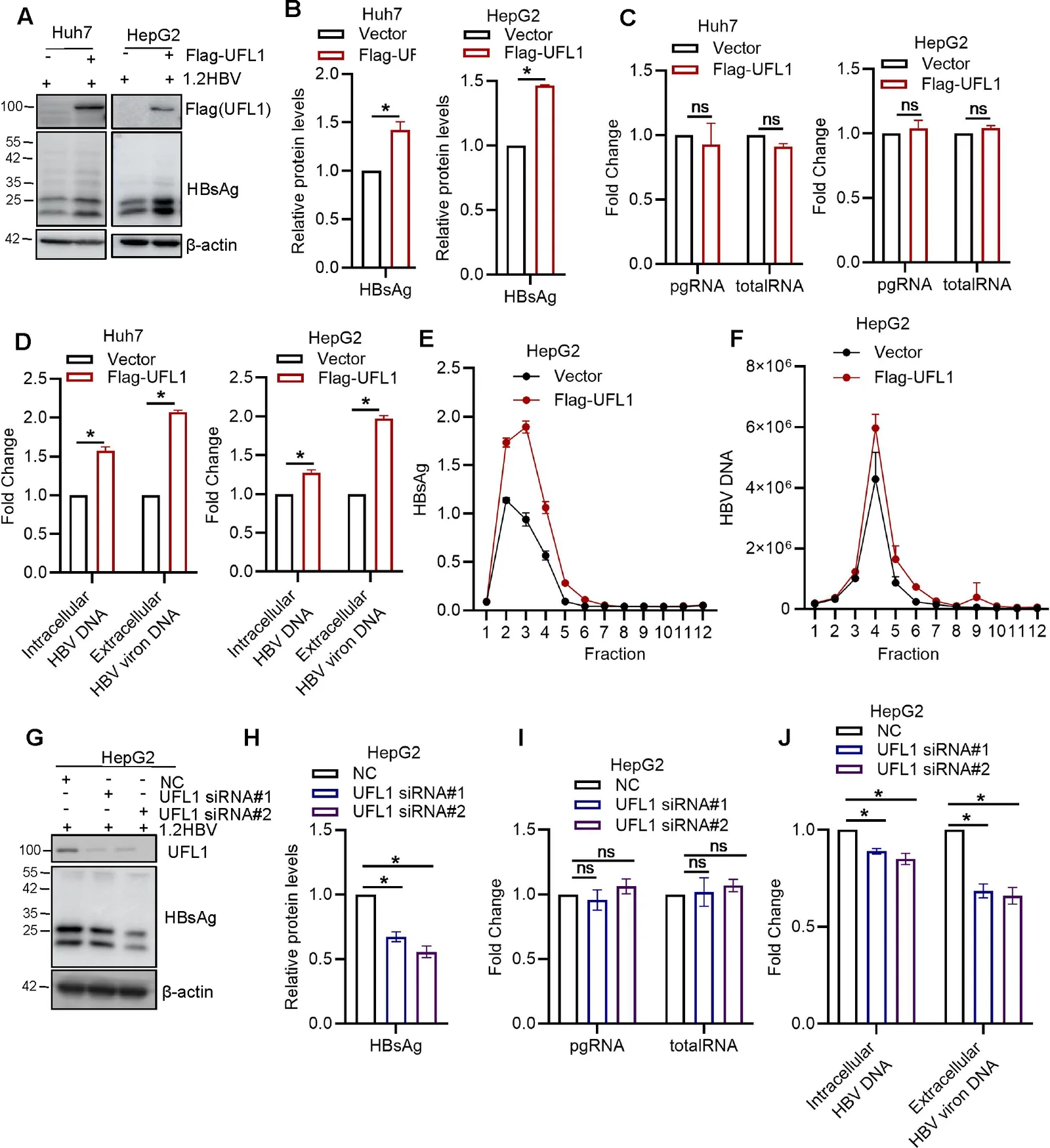
UFL1 modulated HBV virions and viral protein output without altering HBV RNA abundance. (**A–D**) HepG2 cells were co-transfected with 1.2-mer HBV replicon and plasmids encoding Flag-UFL1 (or control vector); intracellular HBsAg was assessed via western blot analysis (**A**), HBsAg in culture supernatants was analyzed by ELISA (**B**), HBV pgRNA and total RNA levels were quantified by reverse transcription-quantitative PCR (RT-qPCR) (**C**), and intracellular capsid-derived HBV DNA and immunoprecipitated extracellular virion DNA were quantified by qPCR (**D**). (**E, F**) Sucrose density gradient ultracentrifugation was performed to separate SVPs and Dane particles, which were quantified by ELISA (**E**) and HBV DNA qPCR (**F**), respectively. (**G–J**) (loss-of-function complement to panels **A–D**). HepG2 cells were co-transfected with 1.2-mer HBV replicon and UFL1-targeting siRNAs. (G) Immunoblot of intracellular HBsAg; (H) ELISA of secreted HBsAg; (I) RT-qPCR of pgRNA and total HBV RNA; (J) qPCR of intracellular core-associated HBV DNA and immunocapture-qPCR of extracellular virion DNA. Data represent ≥3 independent experiments; error bars represent SD; *P < 0.05.

SHBs is crucial for HBV replication and virion production (26). To evaluate the impact of UFL1 on HBsAg and virion production, HepG2 and Huh7 cells were transfected with Flag-UFL1-expressing plasmids and a 1.2-mer HBV replicon. The results demonstrated that UFL1 overexpression increased intracellular HBsAg (mainly SHBs) and secreted HBsAg (Fig. 7A and B), while it did not alter pgRNA or total HBV RNA (Fig. 7C). The results also showed that intracellular capsid-derived and matured virion (immunoprecipitated by anti-HBsAg from the culture supernatant)-derived HBV DNA were elevated upon UFL1 overexpression (Fig. 7D). Density-gradient centrifugation further demonstrated that UFL1 overexpression enhanced both mature virion production and HBsAg-containing SVP release (Fig. 7E and F). Conversely, knockdown of UFL1 had the opposite effects (Fig. 7G through J). Collectively, UFL1 primarily acted post-transcriptionally to increase SHBs and boost virion production.

## DISCUSSION

Chronic hepatitis B remains a major global health burden that progresses through tightly orchestrated steps of viral entry, cccDNA maintenance, nucleocapsid assembly, envelopment, and egress—each dependent on virus–host interfaces (27). Among viral components, the SHBs is central to virion and SVP production and is implicated in disease progression; durable HBsAg loss is therefore a clinical benchmark of “functional cure” (28). Defining host pathways that control SHBs abundance and activity can thus reshape therapeutic strategies toward HBV cure.

Here, using immunoprecipitation-LC-MS/MS, biochemical reconstitution, and functional assays, we identify UFL1—the E3 ligase of the UFMylation—as a direct SHBs interactor that UFMylates nonglycosylated SHBs at Lys122, stabilizes the protein by counteracting TRIM21-mediated ubiquitination, and thereby enhances HBsAg and virion output. Mapping studies localized SHBs binding to the N-terminal 1–212 amino acids of UFL1, consistent with prior reports that key UFMylation components (DDRGK1/UFBP1, UFC1) engage this region (24). UFM1 is conjugated to substrate lysines through a defined pathway: after its C-terminal maturation by UFSP1/2, mature UFM1 (UFM1/ΔC2) is activated by UBA5 (E1), transferred to UFC1 (E2), and finally ligated to targets by the UFL1-DDRGK1 E3 ligase (15). However, a previous study showed that UFMylation of p53 and ASC1 could be detected without UBA5 overexpression, but required other components (UFC1, UFL1, and DDRGK1) (25, 29). Our data reveal that UFMylation components, including UFM1, UBA5, UFC1, and UFL1, are essential for SHBs UFMylation, whereas DDRGK1 is dispensable for this modification. Notably, we observed stronger detection of UFMylated SHBs following MG132 treatment, similar to previous observations for other UFMylation substrates, such as p53 (25). This enhanced signal likely reflects increased substrate stability and reduced proteasomal turnover of UFM1-modified intermediates, allowing their accumulation to detectable levels (11). In addition, proteasome inhibition may induce proteotoxic or ER stress responses that facilitate UFMylation pathway activity (30), thereby further enhancing the detection of SHBs UFMylation. These findings add SHBs to the growing list of UFMylation substrates and place UFL1 at a previously unrecognized node in HBV particle biogenesis.

Mechanistically, our data support a competition model in which UFL1-dependent UFMylation at SHBs Lys122 antagonizes K48-linked ubiquitination and proteasome-dependent turnover, thereby prolonging SHBs half-life. This logic mirrors established roles of UFMylation in stabilizing diverse substrates by opposing ubiquitin-dependent degradation and autophagy (e.g., p53, NLRP3, PD-1) (25, 31, 32). Importantly, in these studies, UFMylation was associated with stabilization of the total substrate protein rather than accumulation of the UFMylated species itself, suggesting that UFMylation functions as a dynamic and reversible regulatory modification (16). Consistent with this model, UFL1 overexpression predominantly increased the steady-state level of non-UFMylated SHBs, suggesting that transient UFMylation is sufficient to protect SHBs from proteasome-mediated degradation. Furthermore, substitution of Lys122 with arginine abolished the ability of UFL1 to stabilize SHBs, confirming that Lys122 is the critical UFMylation site required for this regulatory mechanism. Functionally, UFL1 amplified SHBs-driven phenotypes relevant to liver disease biology—hepatoma cell migration and VEGFA-mediated endothelial tube formation—linking a host conjugation system to pro-migratory and pro-angiogenic outputs. This aligns with the broader paradigm that post-translational modifications tune viral protein oncogenicity (e.g., PRMT5 on KSHV vCyclin; HBx NEDDylation by HDM2) and extends it to UFMylation (33, 34). Given the associations between dysregulated UFMylation and multiple cancers, including hepatocellular carcinoma (35), our data suggest that UFL1-SHBs coupling may contribute to HBV-related pathogenesis by sustaining high SHBs burden and its downstream cellular effects.

At the level of the viral life cycle, UFL1 increased intracellular SHBs and boosted virion production without measurably altering pgRNA or total HBV RNA, indicating a post-transcriptional control point centered on SHBs homeostasis. This complements prior observations in other viruses where UFMylation promotes assembly or immune evasion (e.g., DENV/ZIKV NS2A, NS2B-NS3, and capsid; EBV BILF1-MAVS axis; HAV RPL26) (17). Thus, UFMylation emerges as a versatile regulator of viral particle production across phylogenetically distinct viruses, now including HBV through SHBs stabilization.

In summary, we establish SHBs as, to our knowledge, the first HBV protein substrate of UFMylation and delineate a UFL1-SHBs axis that stabilizes SHBs, augments disease-relevant cellular phenotypes, and enhances HBsAg and virion production. By repositioning UFMylation from a background cellular pathway to a direct regulator of an HBV structural antigen, our findings refine models of HBV particle biogenesis and nominate the UFL1-SHBs interface as a host-directed target to lower HBsAg and advance toward a functional cure.

## MATERIALS AND METHODS

### Cell lines and culture

HepG2, HEK293T, and EA.hy926 human vascular endothelial cells were obtained from ATCC (Manassas, VA, USA), and Huh7 cells from JCRB (Osaka, Japan). Huh7, HEK293T, and EA.hy926 were cultured in Dulbecco’s modified Eagle medium (DMEM; Gibco) and HepG2 in minimum essential medium (MEM; Gibco), each supplemented with 10% heat-inactivated fetal bovine serum (FBS; HyClone). Cells were maintained at 37°C in a humidified 5% CO₂ incubator and passaged at 70%–80% confluence.

### Antibodies and reagents

The primary antibodies used were Anti-UFM1 antibody (Abcam, ab109305), anti-UFL1 Antibody (Novusbio, NBP1-90691), anti-β-Actin Rabbit mAb (Cell Signaling Technology, 4970s), Anti-HBV Surface Antigen Monoclonal antibody (Creative Diagnostics, DMABT-51328MH), anti-DYKDDDDK Tag Rabbit mAb (Cell Signaling Technology, 14793), anti-HA-Tag Rabbit mAb (Cell Signaling Technology, 3724), anti-c-Myc Rabbit mAb (Cell Signaling Technology, 18583), anti-UBA5 antibody (Proteintech, 68537-1-Ig), anti-UFC1 antibody (Proteintech, 15783-1-AP), anti-DDRGK1 antibody (Proteintech, 21445-1-AP). The secondary antibodies used in western blotting and immunofluorescence assays were Anti-rabbit IgG, HRP-linked Antibody (Cell Signaling Technology, 7074), Anti-mouse IgG, HRP-linked Antibody (Cell Signaling Technology, 7076), Anti-mouse IgG (H+L), F(ab′)2 Fragment (Alexa Fluor 488 Conjugate) (Cell Signaling Technology, 4408), Anti-rabbit IgG (H+L), F(ab′)2 Fragment (Alexa Fluor 594 Conjugate) (Cell Signaling Technology, 8889). Reagents used were as follows: Western and IP lysis buffer (Beyotime Biotechnology, P0013), anti-Flag M2 affinity gel (Sigma, A2220), Protein A/G PLUS-Agarose (Santa Cruz, sc-2003), Strep-Tactin XT Superflow high-capacity beads (Iba-Lifesciences, #2-5030-002), Glutathione Sepharose 4B (GE Healthcare, 17-0756-01), the TNT T7 Quick Coupled transcription/translation system (Promega, L1171), isopropyl-β-D-thiogalactoside (IPTG) (Sigma, 367-93-1), Ni-NTA Agarose (QIAGEN, 30210), 4% paraformaldehyde (Beyotime Biotechnology, P0099), DAPI (Beyotime Biotechnology, C1002), Matrigel Basement Membrane Matrix (Corning, 356234), ATP (Beyotime Biotechnology, D7378), MG132 (Med Chem Express, HY-13259), cycloheximide (CHX) (Med Chem Express, HY-12320), Protease inhibitor cocktail (Med Chem Express, HY-K0010), TRIzol reagent (Invitrogen, 15596026), ExScript reverse transcription-PCR kit (Accurate Biology, AG11705), SYBR Premix Ex TaqTM kit (Accurate Biology, AG11701), Hot-start Taq polymerase kit (TaKaRa, RR390A), Hepatitis B Virus Surface Antigen (HBsAg) Detection Kit (ELISA) (Wantai Bio, S10980090), Hepatitis B Virus e Antigen (HBeAg) Diagnostic Kit (ELISA) (Wantai Bio, 201634022327).

### Plasmids

The pRep-1.2HBV (HBV DNA, 3,215 bp, genotype B, adw subtype, GenBank accession number: AF100309) and its empty control of pRep-Sal, the plasmids pCDH-Strep-Flag, pCDNA3.1-SHBs-Flag (the SHBs gene was PCR-amplified from pRep-1.2HBV), pCMVTNT-SHBs-Flag, and pCDNA3.1-HA-K48Ub as described previously (11). pCMVTNT-SHBs/N146Q-Flag, which expresses the SHBs N146Q mutant, was generated by site-directed mutagenesis using pCMVTNT-SHBs-Flag as the template. To generate pCDH-SHBs-Strep-Flag, SHBs was PCR-amplified from pCDNA3.1-SHBs-Flag and inserted into pCDH-Strep-Flag. SHBs lysine mutants (K24R, K122R, K141R, K160R, N146Q) were produced by site-directed mutagenesis and cloned into pCDH-Strep-Flag. UFMylation components expression plasmids, including pCDNA3.1-myc-UFL1, pCDNA3.1-Flag-UFL1, pCDNA3.1-myc-UFM1, pCDNA3.1-UBA5-myc, pCDNA3.1-UFC1-myc, and pCDNA3.1-DDRGK1-myc, were created by cloning UFL1 (GenBank accession number: NM-015323.5), UFM1 (GenBank accession number: NM-016617.4), UBA5 (GenBank accession number: NM-024818.6), UFC1 (GenBank accession number: NM-016406.4), and DDRGK1 (GenBank accession number: NM-023935.3) genes, tagged with myc or Flag, and were synthesized by General Biosystems and inserted into pCDNA3.1/myc-His(−)A (Invitrogen, V80020). pGEX-4T-1-UFL1 and various UFL1 deletion mutants were created by cloning PCR-amplified UFL1 or various UFL1 deletion mutant genes into pGEX-4T-1 (GE Healthcare, 28-9545-49), utilizing pCDNA3.1-myc-UFL1 as templates. To generate pCDNA3.1-myc-UFL△1-212, UFL1△1-212 was PCR-amplified from pGEX-4T-1-UFL1△1-212 and inserted into pCDNA3.1/myc-His(−)A. pET-28a(+)-UFL1, pET-28a(+)-UBA5, pET-28a(+)-UFC1, and pET-28a(+)-DDRGK1 were created by cloning PCR-amplified UFL1, UBA5, UFC1, or DDRGK1 genes into pET-28a(+) (Novagen, 69864). DNA coding for an inactive form of UFM1 with a deletion of three amino acids at the C-terminus (UFM1/ΔC3), tagged with myc, was cloned into pCDNA3.1/myc-His(−)A. DNA coding for the active form of UFM1 with a deletion of two amino acids at the C-terminus (UFM1/ΔC2) was cloned into pET-28a(+).

### RNA interference

Cells were transfected with either a negative control siRNA or two specific siRNAs (designed and synthesized by GenePharma Technology) using the manufacturer’s recommended protocol. After 48 h, cells were harvested, and knockdown efficiency was validated by western blot. The sequences of siRNAs used in this study were NC: 5′-UUCUCCGAACGUGUCACGUTT-3′; UFL1 siRNA#1: 5′-GGAACUUGUUAAUAGCGGATT-3′; UFL1 siRNA#2: 5′-CCAUAACUCUCUGAAUGAATT-3′; DDRGK1 siRNA: 5′-GCACUCAGGACACCAUAAATT-3′.

### Affinity purification and LC-MS/MS

The procedures were as described previously (11). Briefly, Huh7 cells were transfected with either pCDH-SHBs-Strep-Flag or the empty control of pCDH-Strep-Flag for 48 h. Cells were then lysed, and the lysates were subjected to immunoprecipitation overnight at 4°C using Strep-Tactin XT Superflow high-capacity beads. The immunoprecipitated complexes were separated by SDS-PAGE, and the protein bands corresponding to approximately 90 kDa were excised and analyzed by LC-MS/MS on an Easy-nLC 1200 system coupled to an Orbitrap Exploris 480 mass spectrometer (Thermo Fisher Scientific). The resulting MS data were processed with MaxQuant (version 2.1.4.0) and searched against the UniProt Homo sapiens database (https://www.uniprot.org/proteomes/UP000005640) for protein identification.

### Western blot analysis

Cells were lysed on ice for 30 min using Western and IP lysis buffer supplemented with 1 mM PMSF and a protease inhibitor cocktail after 48 h post-transfection. The protein concentration of the lysates was determined using a BCA Protein Assay Kit according to the manufacturer’s instructions. Equal amounts of protein were then separated by 12% SDS-PAGE and transferred onto PVDF membranes. After blocking with 5% BSA in TBST, the membranes were incubated overnight at 4°C with primary antibodies. Following extensive washing with TBST, the membranes were probed with an HRP-conjugated secondary antibody for 1 h at room temperature. Protein bands were finally visualized using the BeyoECL Star chemiluminescent substrate.

### Co-immunoprecipitation

Cellular lysates were prepared, and the soluble protein fraction was incubated overnight with Strep-Tactin XT Superflow high-capacity beads or 4 μg of antibodies and 100 μL of a 50% slurry of protein A agarose at 4°C. After incubation, the beads were extensively washed to remove non-specifically bound proteins. The immunoprecipitated complexes were then separated by SDS-PAGE and analyzed by western blotting to identify interacting partners.

### GST pull-down (*in vitro* binding)

For the GST pull-down assay, GST or GST-fused proteins were expressed in *E. coli* strain Rosetta (DE3) by induction with 0.5 mM IPTG at 16°C for 12 h and purified using glutathione-Sepharose beads. Flag-tagged SHBs proteins were synthesized *in vitro* using the TNT Quick Coupled Transcription/Translation System programmed with the pCMVTNT-SHBs-Flag plasmid. For the binding reaction, the purified GST or GST fusion proteins immobilized on beads were incubated with the *in vitro*-translated SHBs proteins overnight at 4°C. After extensive washing, the bound proteins were eluted, separated by SDS-PAGE, and analyzed by immunoblotting with anti-SHBs antibody.

### Immunofluorescence microscopy

Huh7 cells grown on coverslips were transfected with either pCDH-SHBs-Strep-Flag or the control vector for 48 h. The cells were then washed with PBS, fixed with 4% paraformaldehyde for 10 min, and permeabilized with 0.2% Triton X-100 for 10 min at room temperature. After blocking with 5% BSA for 30 min, the cells were incubated with specific primary antibodies (1:200 dilution) overnight at 4°C, followed by incubation with fluorescently labeled secondary antibodies (1:400 dilution) for 1 h at room temperature in the dark. Cell nuclei were counterstained with DAPI for 5 min. Images were acquired using a Leica SP8 laser scanning confocal microscope.

### Protein half-life assay (CHX chase assay)

Cells were co-transfected with pCDH-SHBs-Strep-Flag together with either pCDNA3.1-Flag-UFL1 or the empty vector for 40 h. Subsequently, CHX, a protein synthesis inhibitor, was added to the culture medium at a final concentration of 200 µg/mL. Cells were harvested at designated time points (0, 30, 60, 90, and 120 min) after CHX addition. The protein lysates were then analyzed by western blotting to monitor the time-dependent degradation of SHBs.

### Ubiquitination assays

HepG2 cells underwent transfection with pCDH-SHBs-Strep-Flag together with pCDNA3.1-HA-K48Ub for a duration of 40 h. Following the transfection period, the cells were treated with 20 μM MG132, a proteasome inhibitor, for 8 h to prevent the degradation of ubiquitinated proteins. Cells were then lysed in Western and IP lysis buffer supplemented with PMSF and a protease inhibitor cocktail. The clarified lysates were subjected to immunoprecipitation using Strep-TactinXT Superflow high-capacity beads to isolate SHBs-Strep-Flag. The immunoprecipitated complexes were analyzed by western blotting.

### *In vivo* and *in vitro* UFMylation assays

#### In vivo

Huh7 and HepG2 cells were co-transfected with pCDH-SHBs-Strep-Flag along with plasmids expressing UFMylation components for 48 h. Subsequently, cells were lysed in Western and IP lysis buffer supplemented with PMSF and a protease inhibitor cocktail for 30 min. After incubation with Strep-TactinXT Superflow overnight at 4°C, the immunoprecipitates were subjected to SDS-PAGE, followed by western blot analysis.

#### In vitro

*In vitro* UFMylation assay was performed as described previously (25). Briefly, His-tagged UFL1, UBA5, UFC1, UFM1/ΔC2, and DDRGK1 were ectopically expressed in *E. coli* strain Rosetta (DE3) and purified using Ni-NTA Agarose. SHBs was purified from HEK293T cells expressing SHBs-Strep fusion protein. His-UBA5 (0.1 µM), His-UFC1 (0.1 µM), His-UFL1, His-DDRGK1 (0.1 µM), His-UFM1/ΔC2 (0.1 µM), and SHBs (0.1 µM) were mixed in a reaction buffer (0.05% BSA, 50 mM HEPES, pH 7.5) containing 5 mM ATP and 10 mM MgCl_2_ and incubated at 30°C for 90 min. The mixtures were denatured by boiling in SDS-PAGE loading buffer containing 5% β-mercaptoethanol for 10 min.

### Transwell migration assays

Cell migration was assessed using a Transwell chamber assay. Briefly, 5 × 10^4^ Huh7 and HepG2 cells, after overnight starvation, were seeded into the top of the coated filters in 300 μL of serum-free medium; 700 μL of DMEM with 10% FBS was placed in the lower chamber to act as a chemoattractant. Vascular Endothelial Cells EA.hy926 cells were seeded into the upper chamber of a transwell in serum-free medium at 3 × 10^4^ cells per well, and the lower chamber contained 10% FBS culture medium harvested from Huh7 or HepG2 cells transfected with pCDH-SHBs-Strep-Flag together with either pCDNA3.1-Flag-UFL1 or UFL1-targeting siRNA. After a 24 or 48 h incubation, non-migrated cells on the upper surface of the membrane were carefully removed with a cotton swab. The migrated cells on the lower surface were fixed, stained with Giemsa solution, and imaged. The number of migrated cells was counted from five random fields per well using a Qimaging Micropublisher 5.0 RTV camera.

### Tube formation assay

The tube formation assay was conducted using EA.hy926 cells, seeded at a density of 1.5 × 10^4^ cells per well in a 96-well plate coated with Matrigel. The EA.hy926 cells were incubated at 37°C for 15 h in the conditioned medium (CM) obtained from Huh7 or HepG2 cells transfected with pCDH-SHBs-Strep-Flag together with either pCDNA3.1-Flag-UFL1 or UFL1-targeting siRNA. Images were taken under a light microscope. For quantitative analysis, the number of tubes was counted and quantified across five low-power fields at 100× magnification.

### RNA extraction and reverse transcription quantitative PCR

Total RNA was extracted using the TRIzol reagent and reverse transcribed to cDNA using the ExScript reverse transcription-PCR kit. Quantitative real-time PCR was performed in the Mx3000P Real-Time PCR System with the SYBR Premix Ex TaqTM kit following the manufacturer’s instructions. The GAPDH gene was used as a reference gene, and relative RNA levels were calculated using the 2^−ΔΔCt^ method. The paired forward and reverse primers were: 5′-GAGTGTGGATTCGCACTCC-3′ (forward) and 5′-GAGGCGAGGGAGTTCTTCT-3′ (reverse) for pgRNA, 5′-TCACCAGCACCATGCAAC-3′ (forward) and 5′-AAGCCACCCAAGGCACAG-3′ (reverse) for total RNA, 5′-TGCACCACCAACTGCTTAGC-3′ (forward) and 5′-AGCTCAGGGATGACCTTGCC-3′ (reverse) for GAPDH.

### ELISA assay

The expression levels of HBsAg in the culture supernatant were measured using the Hepatitis B Virus Surface Antigen (HBsAg) Detection Kit (ELISA), according to the protocols provided by the manufacturer.

### Quantification of virion DNA from culture supernatants

Virion DNA from culture supernatants was quantified by qPCR as previously described (36). Briefly, HBV virions were immunoprecipitated from PEG8000-concentrated supernatants using an anti-HBsAg antibody bound to protein A/G Sepharose beads. The immunoprecipitates were treated with DNase I to remove any contaminating plasmid DNA. Subsequently, viral DNA was released by proteinase K digestion, ethanol-precipitated using glycogen as a carrier, and subjected to quantitative PCR. qPCR was performed using a hot-start Taq polymerase kit with the following primers: HBV-fwd 5′-AATGCCCCTATCCTATCAACACT-3′ and HBV-rev 5′-GAGATTGAGATCTTCTGCGACG-3′, along with a TaqMan probe labeled with FAM at the 5′ end and TAMRA at the 3′ end: 5′-FAM-CCCCTAGAAGAAGAACTCCCTCGCCT-TAMRA-3′ was used for detection. The thermocycling conditions were 95°C for 30 s, followed by 40 cycles of 95°C for 5 s and 60°C for 25 s. Fluorescence (FAM) was measured at the end of each annealing/extension step.

### Quantification of the intracellular capsid-derived HBV DNA

Intracellular capsid-derived HBV DNA was quantified by qPCR as previously described (37). In brief, HBV-producing hepatoma cells were lysed with NP-40 lysis buffer (10 mM Tris-HCl [pH 7.9], 50 mM NaCl, 1 mM EDTA, 0.25% Nonidet P-40, protease inhibitor). The lysates were centrifuged at 14,000 × *g* for 5 min to obtain clarified supernatants. To precipitate the HBV capsids, 125 μL of 35% PEG 8000 in 1.75 M NaCl was added to the supernatant, followed by incubation on ice for 16 h. The pellets collected by centrifugation were resuspended and treated with DNase I (37°C, 30 min) to remove residual plasmid DNA. The capsids were then digested with proteinase K, and the total nucleic acids were extracted and precipitated. Quantitative PCR was performed using a hot-start Taq polymerase kit according to the manufacturer’s instructions, with the primers HBV-fwd 5′-AATGCCCCTATCCTATCAACACT-3′ and HBV-rev 5′-GAGATTGAGATCTTCTGCGACG-3′, along with a TaqMan probe labeled with FAM at the 5′ end and TAMRA at the 3′ end: 5′-FAM-CCCCTAGAAGAAGAACTCCCTCGCCT-TAMRA-3′ was used for detection.

### Sucrose density gradient ultracentrifugation

HBV particles were separated by discontinuous sucrose density gradient ultracentrifugation as previously described, with minor modifications (38). Briefly, HBV particles were concentrated from clarified culture supernatants by PEG 8000 precipitation (6%–8%, overnight, 4°C) and resuspended in PBS. Samples were then loaded onto discontinuous sucrose gradients (15%–60% in TNE buffer) by ultracentrifugation at 70,000 × *g* for 5 h at 8°C. Fractions were collected sequentially from the top of the gradient. Mature virions and subviral particles were analyzed by HBV DNA qPCR and HBsAg ELISA, respectively.

### Statistical analysis

Data are mean ± SD from at least three independent experiments with technical triplicates unless stated. Statistical analyses were performed in SPSS 22.0 (IBM). Two-tailed unpaired Student’s *t*-tests were used for two-group comparisons; χ² tests were used for categorical data where applicable. *P* < 0.05 was considered statistically significant. Effect sizes and exact *P* values are reported in figure legends when appropriate.

## Data Availability

The data sets generated and/or analyzed during the current study are available from the corresponding author on reasonable request.

## SUPPLEMENTAL MATERIAL

Fig. S1 jvi.01034-26-s0001.tif Affinity purification-mass spectrometry screening and identification of SHBs-interacting proteins.

Fig. S2 jvi.01034-26-s0002.tif SHBs UFMylation requires UFMylation components.

Fig. S3 jvi.01034-26-s0003.tif UFMylation components increase SHBs expression.

Supplemental legends jvi.01034-26-s0004.docx Descriptive legends for Fig. S1 to S3.

ASM does not own the copyrights to Supplemental Material that may be linked to, or accessed through, an article. The authors have granted ASM a non-exclusive, world-wide license to publish the Supplemental Material files. Please contact the corresponding author directly for reuse.
