# Supplementary material for: UFMylation of SHBs increases protein stability by antagonizing ubiquitination and promotes virion production
Source: J Virol. 2026 Sep 22;100(10):e01034-26. doi: 10.1128/jvi.01034-26 (PMC13712890; doi:10.1128/jvi.01034-26)
Supplement: Supplementary file 1 [file jvi.01034-26-s0004.docx]

**SUPPLEMENTARY FIGURE LEGENDS**

**Figure S1. Affinity purification–mass spectrometry screening and identification of SHBs-interacting proteins.**

**(A)** Representative MS/MS of a UFL1 tryptic peptide, with annotated b/y fragment ions confirming peptide sequence assignment and protein identification. (**B)** Reciprocal Co-IPs from Huh7 cells co-transfected with plasmid encoding myc-UFL1 and SHBs-Strep, Strep-Tactin or anti-myc pull-downs were immunoblotted for UFL1 and SHBs, respectively. (**C)** Purified GST and GST-fused UFL1 wild type or deletion fragments (Δ1-212, Δ213-452, Δ453-654, and Δ655-794) were subjected to Coomassie Blue staining.

**Figure S2. SHBs UFMylation requires UFMylation components.**

**(A)** HepG2 cells were co-transfected with plasmids encoding SHBs–Strep together with Myc-tagged UFMylation components, UFM1 alone, UFL1 alone, or UBA5/UFC1/UFL1/ DDRGK1. Forty-eight hours post-transfection, SHBs UFMylation levels were assessed. (**B)** HepG2 cells were co-transfected with plasmids encoding SHBs–Strep and Myc-UFM1, together with siRNA targeting DDRGK1. SHBs UFMylation was analyzed by *in vitro* UFMylation assay.

**Figure S3. UFMylation components increase SHBs expression.**

**(A–C)** HepG2 cells were co-transfected with plasmids encoding SHBs–Strep and increasing amounts of UFM1 (A), UBA5/UFC1/UFL1/DDRGK1 (B), or UFM1 together with UBA5/UFC1/UFL1/DDRGK1 (C). Intracellular and secreted HBsAg levels were analyzed by Western blotting and ELISA, respectively. Error bars represent SD; **P* < 0.05.
